# Use of Ceftazidime–Avibactam in a Late-Preterm Newborn with Multidrug-Resistant *Enterobacter hormaechei* Sepsis

**DOI:** 10.3390/antibiotics15070690

**Published:** 2026-07-16

**Authors:** Marcello Trizzino, Veronica Notarbartolo, Luca Pipitò, Gregorio Serra, Bendetta Romanin, Maurizio Carta, Vincenzo Insinga, Maria R. Di Pace, Teresa M. A. Fasciana, Antonio Cascio, Mario Giuffrè

**Affiliations:** 1 Infectious and Tropical Diseases Unit, Sicilian Regional Reference Center for the Fight Against AIDS, AOU Policlinico “P. Giaccone”, 90133 Palermo, Italy; marcello.trizzino@policlinico.pa.it (M.T.); antonio.cascio03@unipa.it (A.C.); 2 Neonatology and Neonatal Intensive Care Unit, AOU Policlinico “P. Giaccone”, 90127 Palermo, Italy; gregorio.serra@unipa.it (G.S.);; 3 Department of Health Promotion, Mother and Child Care, Internal Medicine and Medical Specialties “G. D’Alessandro”, University of Palermo, 90133 Palermo, Italy; 4 Pediatric Surgery Unit, AOU Policlinico “P. Giaccone”, 90127 Palermo, Italy

**Keywords:** neonatal sepsis, *Enterobacter hormaechei*, multidrug resistance, ceftazidime-avibactam, esophageal atresia, NICUs, preterms

## Abstract

**Background:** Neonatal sepsis caused by multidrug-resistant (MDR) Gram-negative pathogens poses significant therapeutic challenges in neonatal intensive care units (NICUs), particularly in surgical neonates. Ceftazidime-avibactam (CAZ-AVI), a novel β-lactam/β-lactamase inhibitor combination, offers activity against extended-spectrum β-lactamases (ESBLs), AmpC, and serine carbapenemases, but neonatal experience remains limited. **Methods and Results:** We present a 35-week preterm neonate with long-gap esophageal atresia who developed late-onset sepsis caused by MDR *Enterobacter hormaechei*, including a KPC-producing carbapenem-resistant isolate, during postoperative recovery. After a multidisciplinary review, the patient received off-label CAZ-AVI at 25 mg/kg/dose (20/5 mg/kg) every 8 h, infused over 2 h, informed by available neonatal pharmacokinetic evidence and weight-adjusted dosing recommendations. Amikacin was administered contextually. Consecutive blood cultures obtained 48–72 h after dual antibiotic therapy initiation became negative and remained sterile, with concordant decline in inflammatory biomarkers, confirming rapid microbiological clearance. Unfortunately, the newborn died in the hours immediately following the final surgery performed to repair the esophageal atresia via end-to-end anastomosis; the cause was complications (hemothorax) unrelated to infectious diseases, although the onset of such severe sepsis may have compromised an already impaired immunological status. **Discussion:** This case shows mechanism-concordant antimicrobial selection and the feasibility of neonatal dosing when MDR *Enterobacterales* drive invasive infection. Our experience supports prospective evaluation of CAZ-AVI in carefully selected septic newborns while emphasizing the importance of rapid carbapenemase genotyping, susceptibility testing, and multidisciplinary oversight.

## 1. Introduction

Neonatal sepsis remains a leading cause of morbidity and mortality worldwide, with particularly high incidence and case fatality in preterm and low-birth-weight infants along with substantial burden within NICUs [1,2,3]. Escalating multidrug resistance among Gram-negative pathogens, especially *Enterobacterales* and *Pseudomonadales* (i.e., *Pseudomonas aeruginosa*), increasingly undermines standard empiric regimens and compels susceptibility-driven, stewardship-aligned therapeutic strategies in NICU settings [2,4,5,6]. Current international and World Health Organization (WHO)-aligned guidance endorses narrow-spectrum combinations such as ampicillin plus gentamicin or vancomycin plus amikacin for initial management, yet acknowledges the evidence gaps and clinical challenges posed by hospital-acquired MDR infections in early life [3,7]. Among the new generation drugs, increasingly used even in neonatal age, there is ceftazidime-avibactam (CAZ-AVI) that combines a third-generation cephalosporin with a non-β-lactam β-lactamase inhibitor active against ESBLs, AmpC, and serine carbapenemases (i.e., KPC and OXA-48) producing microorganisms such as *Enterobacterales* spp., capable of triggering severe infections [6,8,9]. Across adult and pediatric populations aged ≥3 months, CAZ-AVI has demonstrated efficacy in complicated intra-abdominal and urinary tract infections and hospital-acquired/ventilator-associated pneumonia, with pooled analyses showing favorable microbiological responses against β-lactamase-producing pathogens and improved clinical outcomes in selected settings [8,9,10]. In difficult-to-treat bacteremia and nosocomial pneumonia, comparative syntheses report higher clinical cure rates and lower mortality with CAZ-AVI-based therapy than with alternative regimens, reinforcing its role when non-MBL resistance mechanisms predominate [8,9]. Until recently, neonatal use was constrained by the absence of age-specific pharmacokinetic data, but a phase 2a multicenter study supports weight- and age-adjusted regimens for infants < 3 months now, with CAZ-AVI 25 mg/kg/dose (20/5 mg/kg) every 8 h, over 2 h, in both full-term ≤ 28 days of life and preterm neonates from 31 to 44 wks of postmenstrual age (PMA), and a higher 37.5 mg/kg/dose (30/7.5 mg/kg) regimen in full-term neonates > 28 days of life and preterms with PMA > 44 wks, achieving plasma exposures comparable to those observed in older pediatric populations and an acceptable safety profile [11]. This case report describes a late-preterm twin neonate with a long-gap esophageal atresia who developed MDR *Enterobacter hormaechei* sepsis responsive in vitro to CAZ-AVI, and details the off-label dosing, safety monitoring, and multidisciplinary decision-making to inform pragmatic NICU stewardship when susceptibility and clinical context warrant this agent [5,11,12].

## 2. Case Presentation

T. is a male second child born from a first twin pregnancy, monochorionic, diamniotic, at 35 weeks of gestation, resulting in an urgent CT scan due to alterations in the tocographic tracing. On the fourth day of life (DOL), due to a suspected long-gap esophageal atresia, he was admitted to the NICU of the “Paolo Giaccone University Hospital” in Palermo for stabilization and staged surgical management. On the sixth DOL, the newborn underwent gastrostomy and right muscle-sparing thoracotomy with distal tracheoesophageal fistula closure and traction sutures across the long esophageal gap, followed by chest drainage, invasive ventilation, central vascular access, and total parenteral nutrition according to NICU standards for complex foregut anomalies. In the immediate postoperative period, renal indices allowed standard neonatal dosing without reduction (creatinine baseline ~0.52 mg/dL, subsequently ~0.18–0.39 mg/dL), while inflammatory activity was initially mild (C-reactive protein, CRP 3.9 mg/L) before sepsis developed. Within the first week, blood cultures yielded methicillin-resistant *Staphylococcus epidermidis*, successfully treated with vancomycin; the antibiogram had shown vancomycin MIC 1–2 mg/L (S), linezolid 2 mg/L (S), teicoplanin ≤ 2–4 mg/L (S), with resistance to oxacillin (>2 mg/L), gentamicin (>4 mg/L), and several fluoroquinolones, consistent with methicillin-resistant *Staphylococcus epidermidis* (MRSE) bacteremia in a line-exposed neonate. The central venous access was removed and replaced due to suspected CLABSI. Soon after, *Enterobacterales* emerged as the primary concern: a thoracic/device source grew *Enterobacter hormaechei* with ESBL/AmpC phenotype and broad β-lactam resistance, while serial blood cultures isolated *Enterobacter cloacae complex* with blaCTX-M (Cefotaxime-Munich β-lactamase), confirming late-onset Gram-negative sepsis in the postoperative course (13 days after surgery). Also in this case, the thoracic device was removed. Inflammatory biomarkers rose sharply (PCT 13.4–24.3 μg/L, IL-6 up to ~3268 pg/mL, CRP up to 158 mg/L), in parallel with hematologic/coagulation evidence of severe sepsis-associated DIC marked by profound thrombocytopenia (platelets 2–9 × 10^3^/μL), anemia (Hb nadir 6.9 g/dL), very high D-dimer (3.7 × 10^4^ ng/mL FEU), hypofibrinogenemia (97 mg/dL), and severe antithrombin deficiency (7–15%), necessitating intensive supportive and transfusional care (repeated transfusions of packed red blood cells, platelets, fresh frozen plasma and antithrombin-III). Cholestasis and hypoalbuminemia developed (total/direct bilirubin up to 17/9.9 mg/dL; albumin 21–30 g/L), while renal function remained within neonatal range, supporting appropriate antibiotic dosing. Empiric meropenem was initiated/escalated with device reassessment for source control, yet persistent recovery of *Enterobacter hormaechei* from blood and devices indicated ongoing Gram-negative sepsis with ESBL/AmpC mechanisms and concerning carbapenem profiles depending on the isolate and site. Key susceptibility data informing definitive therapy were as follows: blood *E. cloacae* (ESBL/CTX-M) was susceptible to amikacin (≤4 mg/L, S), CAZ-AVI (MIC 0.5/4 mg/L, S), ceftolozane/tazobactam (1/4 mg/L, S), meropenem (≤0.125 mg/L, S), meropenem-vaborbactam (≤2/8 mg/L, S), colistin (≤0.5 mg/L, S), while resistant to most cephalosporins, piperacillin/tazobactam, and fluoroquinolones. A device-associated *Enterobacter hormaechei* isolate showed amikacin ≤ 4 mg/L (S), CAZ-AVI MIC 0.5/4 mg/L (S), ceftolozane/tazobactam 2/4 mg/L (S), meropenem ≤ 0.125 mg/L (S), imipenem 0.5 mg/L (S), colistin ≤ 1 mg/L (S), with resistance to third/fourth-generation cephalosporins, piperacillin/tazobactam, and fluoroquinolones. Critically, a subsequent bloodstream *Enterobacter hormaechei* exhibited a KPC-producing carbapenem-resistant profile with CAZ-AVI MIC 1/4 mg/L (S), amikacin ≤ 4 mg/L (S) and meropenem-vaborbactam ≤ 2/8 mg/L (S), while resistant to meropenem (>16 mg/L), imipenem (>8 mg/L), ertapenem (>1 mg/L), ceftazidime (>16 mg/L), fluoroquinolones, with PCR confirming KPC positive and NDM (new Delhi Metallo-β-lactamase)/OXA-48/VIM (Verona Integron-encoded Metallo-β-lactamase) negative, aligning perfectly with avibactam’s serine carbapenemase inhibition spectrum. In the respiratory tract, endotracheal aspirates repeatedly yielded *Enterobacter hormaechei* susceptible to CAZ-AVI (0.5/4 mg/L, S) and amikacin (≤4 mg/L, S), with variable susceptibility to carbapenems and ceftolozane/tazobactam across sampling episodes, consistent with airway colonization under ventilatory support.

At 25 days of age, after a multidisciplinary review and parental consent, off-label CAZ-AVI was started at 25 mg/kg/dose (20/5 mg/kg) every 8 h, infused over 2 h, according to the available neonatal pharmacokinetic evidence, with renal, hepatic and clinical monitoring throughout. The test results were always within the normal range for the patient’s age. This dose was established according to the gestational and postnatal age of the newborn and it was administered alongside amikacin (typically 15 mg/kg, once daily, in newborns, up to 30 mg/kg, once daily, in children and adults) both to reduce the likelihood of resistance developing with CAZ-AVI monotherapy alone and because of the worsening clinical condition of our newborn.

The decision to use CAZ-AVI rather than colistin was based on the fact that the latter is more difficult to manage in pediatric patients and it is used only as a rescue therapy. Furthermore, compared to meropenem-vaborbactam, CAZ-AVI is associated with lower rates of resistance, while offering virtually identical pharmacological profiles.

Following initiation, consecutive blood cultures obtained 48–72 h apart were negative and remained sterile, while CRP fell from peak values to 20 mg/L and IL-6 normalized, confirming microbiological and inflammatory responses. Surgical re-interventions included an exploratory laparotomy for an acute abdomen that excluded intra-abdominal infection on culture. CAZ-AVI and amikacin were discontinued after 15 days (after 2 consecutive negative blood cultures). The slow clinical and laboratory improvement allowed the patient to undergo a new surgical operation approximately 12 days after the last negative blood culture, to obtain a definitive esophageal termino-terminal reconstruction with drains, focusing on ongoing source control on thoracic and device management while maintaining prophylactic antimicrobial coverage. Unfortunately, the child died in the hours immediately following surgery due to complications (hemothorax) unrelated to infectious diseases. Maybe, the previously compromised immune status caused a complication during the procedure.

**Figure 1:**
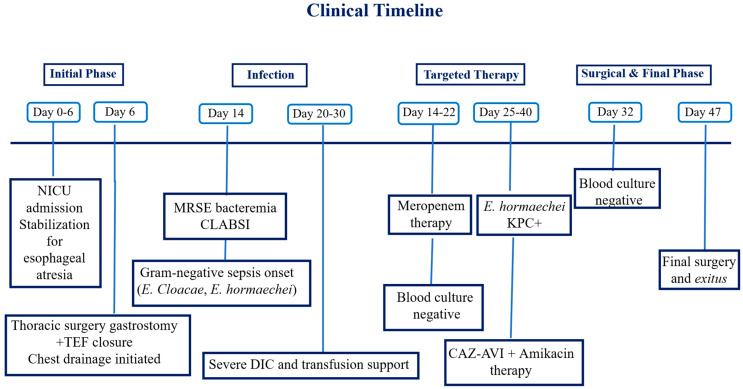
Clinical timeline of MDR Gram-negative sepsis in a 35-week preterm neonate with long-gap esophageal atresia post-thoracotomy. TEF: tracheoesophageal fistula; CLABSI: central line-associated bloodstream infection; DIC: disseminated intravascular coagulation.

The clinical course, microbiological findings, and antimicrobial and surgical management are summarized in Figure 1.

Microbiological isolates and antimicrobial susceptibility are shown in Table 1.

Spectrum of in vitro activity of antibiotics against *Enterobacter* strains is shown in Table 2.

## 3. Discussion

This case illustrates three converging elements that are rarely documented together in neonates: complex foregut surgery with long-gap esophageal atresia, multidrug-resistant *Enterobacterales* sepsis including KPC-producing *Enterobacter hormaechei*, and rapid bloodstream sterilization after a dual antibiotic therapy with Amikacin and CAZ-AVI, consistent with neonatal exposure targets for time-dependent β-lactams [5,6,11,12,13]. The organism’s resistance profile aligned with avibactam’s spectrum against ESBLs/AmpC and serine carbapenemases (KPC/OXA-48), with explicit absence of metallo-β-lactamases, supporting a mechanism-concordant selection that is central to stewardship in the NICU setting [6,8,9]. Amikacin (15 mg/kg IV once daily) was administered alongside ceftazidime-avibactam in consideration of the very severe and rapidly worsening clinical conditions of the child, despite recent scoping reviews suggest comparable microbiological and clinical outcomes between ceftazidime-avibactam monotherapy and combination regimens against CRE, without evidence of superior efficacy for the latter [14,15]. Nevertheless, two consecutive blood cultures turned negative within 48–72 h of dual antibiotic therapy initiation and remained sterile thereafter, paralleled by falling CRP and IL-6 levels, meeting primary microbiological endpoints despite the patient’s subsequent noninfectious demise from hemorrhagic DIC after definitive reconstruction [8,9], as documented in neonatal cases of extensively drug-resistant and pan-resistant *Klebsiella pneumoniae* [16]. The dosing and infusion strategy of CAZ-AVI reflects neonatal dosing recommendations from the first multicenter study, which demonstrated that 25 mg/kg/dose (20/5 mg/kg) every 8 h, infused over 2 h, in both full-term ≤ 28 days of life and preterm neonates from 31 to 44 wks of postmenstrual age (PMA), and a higher 37.5 mg/kg/dose (30/7.5 mg/kg) regimen in full-term neonates > 28 days of life and preterms with PMA > 44 wks, provides a rational template for neonatal bloodstream infections [11]. This data is consistent with successful off-label CAZ-AVI use reported in premature infants with multidrug-resistant *Klebsiella pneumoniae* infection [15] and pandrug-resistant infections [17]. Within older pediatric and adult evidence, pooled phase 3 analyses and a focused meta-analysis in bacteremia and nosocomial pneumonia show that CAZ-AVI delivers robust microbiological responses against non-MBL β-lactamase producers and it is associated with improved clinical cure and reduced mortality in bacteremia compared with alternative regimens, which strengthens confidence when translating to carefully selected neonatal cases with matching mechanisms and susceptibilities [8,9,10]. In this infant, alternative actives (e.g., meropenem-vaborbactam, ceftolozane-tazobactam, colistin) were documented in vitro, but CAZ-AVI was prioritized for direct KPC coverage, β-lactam safety profile, and neonatal feasibility, reserving nephrotoxic or less characterized options as contingency, while ensuring device management and source control proceeded in parallel [5,6]. Two stewardship points are salient for NICUs: first, rapid carbapenemase genotyping (using molecular methods) combined with EUCAST-interpreted MICs (v. 15.0) can pivot management toward avibactam-containing regimens when KPC or OXA-48 are present, and MBLs are excluded; second, prolonged infusion coupled with preserved renal function helps secure pharmacodynamic exposure without dose reduction, particularly critical in high-inoculum postoperative sepsis [5,6,11,12]. The sustained airway colonization by *Enterobacter hormaechei* without relapse to bacteremia under CAZ-AVI underscores that ventilator-associated reservoirs may persist despite bloodstream clearance, reinforcing device care bundles and selective culture surveillance while avoiding reflex broadening in the absence of invasive infection. Importantly, early negative blood cultures should be read with context: in this case, biomarker convergence (CRP and IL-6 decline), negative serial blood cultures, and lack of peritoneal growth after laparotomy triangulated microbiological success despite persistent nonbacteremic morbidity from surgical and coagulopathic complications. The case also illustrates the evolving therapeutic landscape in neonatal MDR sepsis, where conventional empiric regimens are increasingly undermined by ESBL and CRE epidemiology; expert reviews and WHO-aligned guidance emphasize susceptibility-driven escalation and the judicious incorporation of newer β-lactam/β-lactamase inhibitors in sentinel situations, an approach mirrored here by mechanism-matched CAZ-AVI with documented clinical and laboratory response [3,5,6,7]. While single-patient inference is limited and confounded by concurrent surgeries, the temporal association between appropriately dosed CAZ-AVI and bloodstream sterilization, together with corroborating clinical literature and adult/pediatric outcome syntheses, offers pragmatic evidence supporting its off-label use when non-MBL *Enterobacterales* drive neonatal sepsis [8,9,11,12] and alternatives are compromised or less suitable for neonatal use and safety. Nevertheless, the concomitant administration of amikacin makes it impossible to definitively attribute the successful resolution of the sepsis to CAZ-AVI alone.

Ethical and regulatory guardrails remain essential—documented multidisciplinary review, parental informed consent, and adherence to local stewardship policies—particularly given that neonatal datasets are still maturing and treatment-emergent resistance to advanced inhibitors, though uncommon, has been reported in older cohorts. Practice implications include a simple NICU algorithm: confirm mechanism (exclude MBL), verify CAZ-AVI susceptibility with EUCAST MICs, start the correct dosage of the drug based on the gestational and postmenstrual age of the newborn [11] with renal-adjusted considerations, optimize device and source control, and trend paired microbiology with CRP and IL-6 to document concordant response while planning de-escalation or cessation on clearance and clinical stability [5,11,12,18].

Unfortunately, the newborn died in the hours immediately following the final surgery performed to repair the esophageal atresia via end-to-end anastomosis; the cause was complications (hemothorax) unrelated to infectious diseases, although the onset of such severe sepsis may have compromised an already impaired immunological status. In particular, the previous state of coagulopathy caused by the infection certainly could have predisposed the newborn to bleeding risk.

## 4. Materials and Methods

We present a case report that describes a late-preterm twin neonate with a long-gap esophageal atresia who developed MDR *Enterobacter hormaechei* sepsis responsive in vitro to CAZ-AVI, and details the off-label dosing, safety monitoring, and multidisciplinary decision-making to inform pragmatic NICU stewardship when susceptibility and clinical context warrant this agent. Serial culture tests were performed to determine the most appropriate and specific antibiotic therapy, initially as monotherapy and subsequently as dual therapy. In particular, the highly accurate BioFire FilmArray (Salt Lake City, UT, USA) was used to obtain the fastest possible results for blood cultures. Informed consent was obtained from the parents of the child involved in the study. The ethics committee approved the off-label use of the drugs.

## 5. Conclusions

This experience complements neonatal clinical data by adding real-world, mechanism-aligned bacteremia clearance in a <28-day infant, encouraging prospective registries and targeted trials to refine dosing, infusion strategies, and comparative effectiveness against other novel agents in the neonatal MDR sepsis domain. Further studies are, then, warranted.

## Figures and Tables

**Table 1:** Microbiological isolates and antimicrobial susceptibility.

Specimen	Microrganism	Susceptible to Antibiotics MICs (mg/L)	Resistant to Antibiotics MICs (mg/L)
blood (DOL 9)	*Staphylococcus epidermidis*	Daptomycin **0.5** Linezolid **2** Teicoplanin **4** Tigecycline **0.25** Trimethoprim/Sulfamethoxazole **≤ 0.5/9.5** Vancomycin **2**	Clindamycin **> 1** Gentamicin **> 4** Oxacillin **> 2**
blood (DOL 14)	*Staphylococcus epidermidis*	Daptomycin **1** Clindamycin **≤ 0.25** Linezolid **2** Teicoplanin **≤ 2** Tigecycline **0.5** Trimethoprim/Sulfamethoxazole **≤ 0.5/9.5** Vancomycin **1**	Gentamicin **> 4** Oxacillin **> 2**
catheter/chest drain (DOL 14)	*Enterobacter hormaechei* (ESBL/AmpC)	Amikacin **≤ 4** Ceftazidime-avibactam **0.5/4** Ceftolozane-tazobactam **2/4** Colistin ≤ **1** Meropenem ≤ **0.125**	Ampicillin > **16** Cefotaxime > **4** Ceftazidime > **16** Gentamicin **> 4** Piperacillin-tazobactam **32/4** Trimethoprim/Sulfamethoxazole **> 8/152**
blood (DOL 19)	*Enterobacter cloacae* (ESBL/CTX-M)	Amikacin **≤ 4** Ceftazidime-avibactam **0.5/4** Ceftolozane-tazobactam **1/4** Colistin ≤ **0.5** Meropenem-vaborbactam ≤ **2/8** Meropenem ≤ **0.125**	Ampicillin > **8** Ceftazidime > **16** Ciprofloxacin > **1** Gentamicin **> 4** Piperacillin-tazobactam **32/4** Trimethoprim/Sulfamethoxazole **> 4/76**
central venous catheter (DOL 20)	*Enterobacter cloacae* (ESBL/CTX-M)	the same as above	the same as above
blood (DOL 22)	negative		
blood (DOL 25)	*Enterobacter hormaechei* (KPC-producing CRE)	Amikacin **≤ 4** Ceftazidime-avibactam **1/4** Colistin **1** Meropenem-vaborbactam ≤ **2/8**	Ampicillin > **8** Ceftazidime > **16** Ceftolozane-tazobactam > **4/4** Ciprofloxacin > **1** Gentamicin **> 4** Meropenem > **16** Piperacillin-tazobactam > **64/4** Trimethoprim/Sulfamethoxazole **> 4/76**
blood (DOL 25)	*Enterobacter hormaechei* (KPC-producing CRE)	the same as above	the same as above
endotracheal tube (DOL 32)	*Enterobacter hormaechei* (ESBL)	Amikacin **≤ 4** Ceftazidime-avibactam **0.5/4** Ceftolozane-tazobactam ≤ **0.5/4** Meropenem-vaborbactam ≤ **2/8** Meropenem ≤ **0.125**	Ampicillin > **8** Ceftazidime **16** Ciprofloxacin > **1** Gentamicin **> 4** Piperacillin-tazobactam **16/4** Trimethoprim/Sulfamethoxazole **> 4/76**
blood (DOL 32)	negative		
central venous catheter (DOL 32)	negative		
blood (DOL 35)	negative		

DOL: day of life.

**Table 2:** Spectrum of in vitro activity of antibiotics against *Enterobacter* strains isolated from the patient. R, resistant; S, susceptible; -, Not tested.

Antibiotic Class	*E. cloacae* (ESBL/CTX-M)	*E. hormaechei* (ESBL/AmpC)	*E. hormaechei* (KPC-CRE)
3rd/4th Gen Cephalosporins	R	R	R
Ceftazidime-Avibactam (CAZ-AVI)	S	S	S
Ceftolozane-Tazobactam (C/T)	S	R	-
Meropenem	S	S	R
Meropenem-Vaborbactam	S	-	S
Carbapenems (Imi/Ert)	S	S	R
Piperacillin-Tazobactam	R	R	R
Fluoroquinolones	R	R	R
Colistin	S	S	S
Gentamicin	-	R	R
Amikacin	S	S	S

## Data Availability

Data supporting reported results can be found on Pubmed research.
